# Oral Health Status and Risk Factors for Caries in Permanent Teeth among 12-year-old Students in Guangdong, Southern China: A Population-based Epidemiological Survey

**DOI:** 10.3290/j.ohpd.a45076

**Published:** 2020-09-04

**Authors:** Linmei Wu, Jianbo Li, Yanli Zhang, Yueshan Zhou, Yihao Liang, Shaohong Huang

**Affiliations:** a Dentist, Stomatological Hospital, Southern Medical University, City of Guangzhou, Guangdong, China. Performed the statistical analysis, drafted and wrote the manuscript.; b Dentist, Dental Disease Prevention and Treatment Center of Guangdong Province, Stomatological Hospital, Southern Medical University, City of Guangzhou, Guangdong, China. Wrote the manuscript.; c Professor, Dental Disease Prevention and Treatment Center of Guangdong Province, Stomatological Hospital, Southern Medical University, City of Guangzhou, Guangdong, China. Study design, wrote the manuscript.

**Keywords:** caries, permanent teeth, risk factors, students

## Abstract

**Purpose:**

To assess the current prevalence of caries, gingival bleeding, calculus, and fluorosis and to determine the risk factors for caries in permanent teeth of 12-year-old students in Guangdong Province, China.

**Materials and Methods:**

A cross-sectional survey was carried out among 1920 12-year-old students in Guangdong Province. All participants were required to undergo an oral health examination. Information concerning the subjects’ family background, dietary habits, and relevant oral health behaviour were collected in a structured questionnaire. Chi-squared tests, Wilcoxon rank-sum tests, and the logistic regression model were used in the statistical analysis.

**Results:**

The prevalence rates of caries in permanent teeth, gingival bleeding, calculus, and fluorosis were 43.07%, 40.57%, 43.75%, and 5.05%, respectively; the average decayed, missing, and filled teeth (DMFT) index was 1.06±1.721. Residence (odds ratios [OR] rural=1.798, 95% CI: 1.485-2.177), sex (OR female=1.352, 95% CI: 1.121-1.631), paternal education level (OR >9 years=0.755, 95% CI: 0.608-0.936), presence of calculus (OR yes=1.279, 95% CI: 1.057-1.548), and frequency of consumption of sugary snacks (OR frequently=1.418, 95% CI: 1.064-1.890) were statistically significantly associated with the risk of permanent teeth caries.

**Conclusion:**

Oral health in 12-year-olds in Guangdong Province remains to be improved. Rural residence, female sex, the presence of calculus, and frequent consumption of sugary snacks could increase the risk of caries. The paternal educational level was negatively associated with the risk of caries, whereas we found no association between the maternal educational level and the risk of caries in this population.

Oral diseases were one of the most common disorders in the non-fatal health estimates Global Burden of Diseases (GBD) cause hierarchy in 2017.^15^ As the most common oral disease among students, dental caries has been associated with deleterious impacts on general health and well-being, as they have been associated with pain, poor school performance, poor quality of life and an economic burden on the family.^2,7,18^ Moreover, despite being a branch of general health, oral health is often disregarded by the public, particularly in low- and middle-income countries.^3^ Thus, oral health remains a long-term challenge, and additional efforts are needed, especially in developing countries.

The prevalence of oral diseases is dynamic. Accordingly, large-scale oral epidemiological surveys are necessary for oral health status surveillance every ten years. Decennial surveys remain important for researchers to elucidate the current epidemic characteristics and risk factors for oral disease. Moreover, data collected according to the World Health Organization (WHO) criteria can be used for comparisons between regions and countries. Most importantly, the results provide scientific evidence for the development of prevention strategies by dental public health practitioners and practising dentists.

The prevalence and development of oral diseases vary among regions. Since 1970, a continuous downward trend in caries in permanent teeth in children in the USA has been detected.^34^ Similarly, in England, caries prevalence decreased from 72% to 41% in 5-year-olds and from 97% to 46% in 15-year-olds between 1973 and 2013.^27^ However, among 12-year-olds in China, caries prevalence and the DMFT index increased from 28.9% to 34.5% and 0.54 to 0.86, respectively, from 2005-2015.^33,36^

In recent years, rapid growth has occurred in Guangdong Province, affecting dietary habits, oral health behaviours, demographics, and economic development. These changes influence a population’s oral health. According to the Sixth National Census, the population of Guangdong Province has exceeded 100 million people, making it the largest province in China, and the urban population is increasing. Additionally, the gross domestic product (GDP) of Guangdong reached 1.024 trillion USD, increasing 291.3-fold from 1980 to 2015, ranking it the highest in China for the past thirty years. Dietary patterns of many Chinese individuals have transitioned from a traditional diet to a modern diet, with high intake rates of sugar and energy-dense foods.^39^ However, the current oral health status and its associated risk factors in 12-year-old students in Guangdong Province were unknown before the present survey.

Therefore, this purpose of this study was to conduct an epidemiological survey on the oral health status in a representative population of 12-year-old students in 2015-2016 to identify the prevalence of oral diseases and determine their associated risk factors in Guangdong Province.

## Materials and Methods

### Ethical Consideration

The Oral Health Survey scheme was approved by the Stomatological Ethics Committee of the Chinese Stomatological Association (Permit Number: 2014-003), and all of the parents of the respondents signed informed consent forms.

### Sampling Design

**Fig 1:**
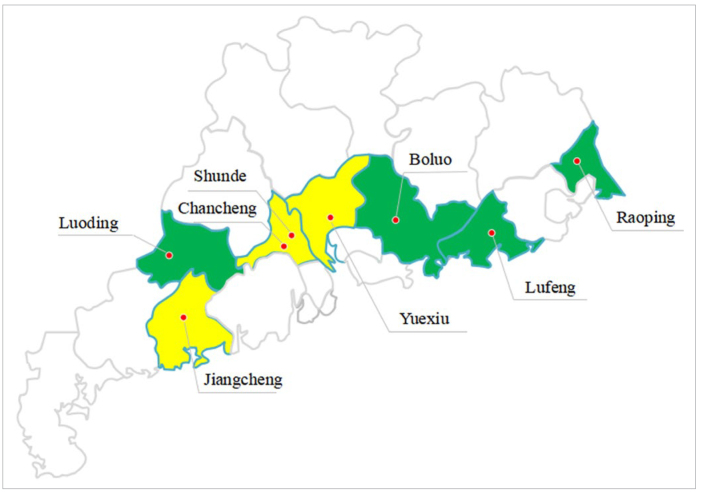
The sampled locations in Guangdong Province, China.

**Fig 2:**
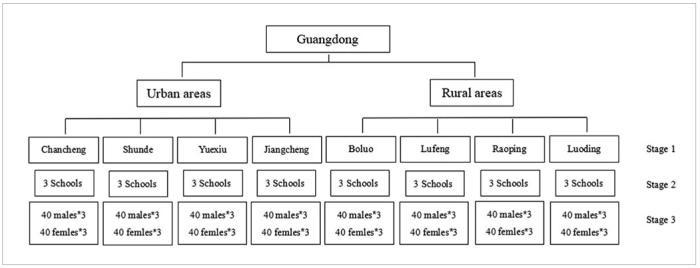
The sampling process.

A stratified, multistage random sampling design was used to establish equal-sized groups of 12-year-old participants who were representative of the province’s population. In the first stage, 4 districts and 4 counties that represented central, eastern, western, and northern Guangdong Province were randomly selected from each stratum using the probability-proportional-to-size (PPS) method with varied population sizes (Fig 1). In the second stage, 3 junior middle schools in each district or county were randomly selected using the PPS method. In the last stage, 80 12-year-old students were recruited from each school. The ratio of male to female participants from each school was 1:1. There were 1920 students in the final sample (Fig 2).

The required sample size was calculated based on the following formula: N = deffµ $\begin{array}{c} 2\\ \alpha /2 \end{array}$p(1-p)/δ2, where ‘deff’ was the sampling design efficiency; ‘α’ was the selected confidence level for statistical testing, ‘p’ was the expected prevalence, and ‘δ’ was the allowable error. Considering urban-rural and male-female stratification, ‘deff’ was set to 4.5 and ‘α’ was set to 0.05, resulting in µ^α/2^ equalling 1.96. The prevalence (p = 28.9%) of caries in children was estimated based on the prevalence of caries in previous national oral health surveys. A theoretical sample size of at least 1891 participants was originally calculated. For ease of grouping and implementation, the actual sample size was adjusted to 1920.

### Clinical Examination

Clinical assessment included an examination to detect dental caries, gingival bleeding, calculus presence, and dental fluorosis. A special investigation team, including 3 oral physicians (a dentist with more than two years of clinical experience and a license for medical practitioners) and 3 recorders who had been engaged in clinical work for more than 3 years and had passed the training conducted by the Technical Guidance Group of National Oral Health Epidemiological Investigation, was established to complete all the on-site investigations. Examinations considering the WHO criteria were performed in a mobile dental chair under artificial light using dental mirrors and community periodontal index (CPI) probes.^37^

### Questionnaire Survey

All participants were asked to complete a structured questionnaire during the clinical examination. The questionnaire included 22 questions regarding their family background, oral behaviours, knowledge, attitudes, and dietary habits related to oral health and their dental experience as well as a self-assessment of general and oral health. The items on the questionnaire have been described in detail elsewhere.^17^ The participants were divided into multiple groups to be examined. An investigator administered the questionnaire to each student by reading each question to the student in the classroom; to avoid influencing the student’s answers, the investigator did not elaborate on the meaning of the questions. The educational levels of the participants’ parents were categorised as >9 years or ≤9 years. Oral health knowledge and attitude scores were computed by summing the total number of correct or positive replies.

### Quality Control

Before the survey, 3 examiners were provided training in theoretical and clinical knowledge by a standard examiner, and the standard examiner reviewed the data of 20 participants who were assessed by the other examiners until the kappa values used to determine inter-examiner reproducibility were >0.85. Moreover, duplicate examinations were randomly conducted for 5% of the participants, and the results of sets of 2 examiners were compared during the survey. The intra-examiner kappa values were 0.90 (examiner 1 to examiner 2), 0.88 (examiner 2 to examiner 3), and 0.88 (examiner 3 to examiner 1).

### Statistical Analysis

To reduce any data entry-related errors, we performed structured double data entry using EpiData version 3.0 (EpiData Association; Odense, Denmark) and applied validation and correction methods. All statistical analyses were performed using IBM SPSS Statistics version 25.0 (IBM; Armonk, NY, USA). The prevalence of caries, gingival bleeding, calculus, and the mean DMFT scores were compared between different areas of residence, sexes, and families using chi-squared tests and Wilcoxon rank-sum tests. The factors related to caries in permanent teeth were analysed by unconditional univariate logistic regression and multivariate logistic regression analyses. Accordingly, a forward stepwise multivariate logistic regression model was applied to examine the risk of dental caries in relation to the independent variables whose p-value was < 0.15 in the bivariate analyses. Statistical significance was set at p < 0.05.

## Results

### Oral Health Status

In this study, the mean DMFT was 1.06 ± 1.721, and the prevalence of permanent teeth caries in 12-year-old students was 43.1%. The prevalence rates of caries were 34.5% and 45.5% in only children and children with siblings, respectively. Statistically significant differences were identified in caries prevalence by sex, residence location, and family type. Additionally, the prevalence of dental fluorosis was 5.1%, the community fluorosis index (CFI) was 0.11, and the popular status according to the Dean Index (DI) was negative (Table 1).

**Table 1:** The oral health status distribution of 12-year-old students in Guangdong Province by residence areas, sex and family type

Demographics	N	DMFT		DMFT>0		Gingival bleeding		Calculus		Fluorosis	
		x/s	Z	n/%	*χ* ^2^	n/%	*χ* ^2^	n/%	*χ* ^2^	n/%	*χ* ^2^
Total	1920	1.06/1.721		827/43.07		779/40.57		840/43.75		97/5.05	
Residence location			*-6.956		*12.594		0.542		0.261		11.824
Urban	960	0.81/1.516		344/35.83		428/44.58		384/49.29		65/6.77*	
Rural	960	1.30/1.873		484/50.31		412/42.91		395/41.15		32/3.33	
Sex			*-4.103		*41.04		0.624		0.135		2.443
Male	960	0.87/1.452		375/45.34		398/44.17		424/41.46		41/4.27	
Female	960	1.25/1.935		452/54.66		381/43.33		416/39.69		56/5.83	
Single-child family			*-4.241		*16.025		0.026		0.145		*4.136
Yes	415	0.76/1.358		143/34.46		183/41.10		165/39.76		29/6.99	
No	1505	1.14/1.800		684/45.45		657/43.65		614/40.80		68/4.52	

*p < 0.001; *χ*^2^: chi-squared.

The results of univariate conditional logistic regression were as follows: No statistically significant relationships were found between the risk of caries in permanent teeth and oral health behaviours.

### Family Background

Family type and parental educational level were included in family background factors in this study. A total of 49.3% and 57.6% of the students’ fathers and mothers had attended school for no more than nine years, which indicated that approximately half of the students had parents with a lower educational status. Statistically significant relationships were found between the caries risk of permanent teeth and single-child family and parental educational level (Table 2).

**Table 2:** The relationship between the risk of permanent teeth caries and family background among 12-year-old students in Guangdong Province

Variables	n/N %	DMFT>0 n (%)	OR	95% CI	p-value^b^
Residence location					
Urban^a^	50	344 (35.83)			< 0.001
Rural	50	483 (50.31)	1.813	1.510-2.177	
Sex					
Male^a^	50	375 (39.06)			< 0.001
Female	50	452 (47.08)	1.388	1.158-1.664	
Single-child family					
Yes^a^	21.61	143 (34.46)			< 0.001
No	78.39	684 (45.45)	1.585	1.264-1.987	
Paternal educational level					
≤9 years^a^	49.32	443 (46.78)			
>9 years	33.33	240 (37.50)	0.683	0.556-0.838	< 0.001
Unknown	17.34	144 (43.24)	0.867	0.674-1.115	0.266
Maternal educational level					
≤9 years^a^	57.55	500 (45.25)			
>9 years	25.94	187 (37.55)	0.728	0.586-0.904	0.004
Unknown	16.51	140 (44.16)	0.957	0.774-1.231	0.732

^a^Reference category; ^b^binary logistic regression.

### Periodontal Condition and Fluorosis

The periodontal condition in 12-year-olds was assessed by gingival bleeding and the presence of calculus. A total of 43.8% and 40.6% of the students had gingival bleeding and calculus, which indicated that half of the students had poor oral hygiene. A statistically significant relationship was found only between the risk of caries in permanent teeth and the presence of calculus (Table 3).

**Table 3:** The relationship between the risk of permanent teeth caries and periodontal conditions and fluorosis among 12-year-old students in Guangdong Province

Variables	n/N %	DMFT>0 n (%)	OR	95% CI	p-value^b^
Gingival bleeding					
No^a^	56.25	471 (43.61)			
Yes	43.75	356 (42.38)	0.951	0.793-1.141	0.589
Calculus					
No^a^	59.43	463 (40.58)			
Yes	40.57	364 (46.73)	1.284	1.069-1.544	0.008
Fluorosis					
No^a^	94.95	789 (43.28)			
Yes	5.05	38 (39.18)	0.951	0.793-1.141	0.589

^a^Reference category; ^b^binary logistic regression.

### Oral Health Behaviour

Approximately half of the students (47.7%) brushed their teeth at least twice per day. Students who were unaware of fluoride toothpaste accounted for 82.8%, and the utilisation rate of oral health care tools, including dental floss (10.1%) and fluoride toothpaste (7.9%), was low. The indices are shown in Table 4.

**Table 4:** The relationship between the risk of permanent teeth caries and oral health behaviours among 12-year-old students in Guangdong Province

Variables	n/N %	DMFT>0 n (%)	OR	95% CI	p-value^b^
Toothbrushing frequency					
≥ twice per day^a^	47.66	402 (43.93)			
≤ once per day	50.05	409 (42.56)	0.946	0.788-1.135	0.548
Fluoride toothpaste use					
Yes^a^	7.86	64 (42.38)			
No	6.35	40 (32.79)	0.663	0.403-1.090	0.105
Unknown	82.81	703 (44.21)	1.077	0.769-1.510	0.665
Dental floss use					
No^a^	89.90	742 (42.99)			
Yes	10.10	85 (43.81)	0.967	0.717-1.304	0.826

^a^Reference category; ^b^binary logistic regression.

### Oral Health Knowledge, Education, and Attitudes

An oral health knowledge score ranged from 0 to 8, with a higher score indicating better oral health knowledge. The oral health attitude score ranged from 0 to 4, with a higher score indicating a more positive attitude. The mean scores of oral knowledge and attitude among the students were 4.54 ± 2.092 and 3.39 ± 0.849, respectively. Among the 12-year-olds, 22.0% agreed that pit and fissure sealants could prevent caries, and approximately half of the students agreed that fluoride toothpaste helps prevent caries. Moreover, only 6.35% students had obtained oral health education. Statistically significant relationships were found between the risk of caries in permanent teeth and oral health knowledge (Table 5, Table 5-1, Table 5-2).

**Table 5:** The relationship between the risk of permanent teeth caries and oral health knowledge and attitudes among 12-year-old students in Guangdong Province

Variables	n/N %	DMFT>0 N (%)	OR	95% CI	p-value^b^
Oral health knowledge					
Score≤4^a^	57.45	513 (45.12)			
Score>4	39.58	314 (40.10)	0.946	0.788-1.135	0.029
Oral health education					
Yes^a^	6.35	42 (34.43)			
No	89.53	759 (44.15)	1.744	0.980-3.105	0.059
Unknown	4.11	49 (62.03)	0.768	0.531-1.109	0.159
Oral health attitude					
Score=4^a^	56.25	374 (44.52)			
Score<4	43.75	453 (41.94)	1.111	0.926-1.333	0.258

^a^Reference category; ^b^binary logistic regression.

**Table 5-1:** Oral health knowledge among 12-year-old students in Guangdong Province

Oral health knowledge	Agree %	Disagree %	Unknown %
Is gum bleeding normal when brushing teeth?	14.32	67.81	17.86
Is gum infection caused by bacteria?	68.18	5.68	26.15
Is brushing the teeth useless in preventing gum infection?	5.68	71.41	22.92
Are dental caries caused by bacteria?	50.05	10.21	39.69
Are dental caries caused by sugar?	72.55	7.19	20.26
Is fluoride useless for tooth protection?	5.42	45.00	49.58
Does pit and fissure sealant protect the teeth?	22.03	12.92	65.05
Is general health influenced by oral diseases?	57.24	10.94	31.82

**Table 5-2:** Oral health attitudes among 12-year-old students in Guangdong Province

Oral health attitudes	Agree %	Disagree %	Indifferent %	Unknown %
Oral health is important for the quality of life	94.38	0.57	2.29	2.76
Regular dental inspection is necessary	64.53	3.80	15.05	16.61
Dental health is decided at birth and is not related to self-care	3.39	90.00	1.20	5.42
Individual effort is important to prevent dental problems	90.57	2.71	1.51	5.21

### Dietary Habits

The frequency of consumption was classified as follows: rarely; sometimes = less than once per day; or frequently = at least once a day. A total of 62.6%, 44.8%, and 43.6% of students were reported to frequently consume snacks, soft drinks, and milk with sugar, respectively. No statistically significant relationships were found between the risk of caries in permanent teeth and dietay habits (Table 6).

**Table 6:** The relationship between the risk of permanent teeth caries and dietary habits among 12-year-old students in Guangdong Province

Dietary habits	n/N %	DMFT>0 n (%)	OR	95% CI	p-value^b^
Snack frequency					
Seldom^a^	13.75	103 (12.45)			
Sometimes	26.41	206 (24.91)	1.070	0.789-1.450	0.664
Frequently	59.84	518 (62.64)	1.283	0.976-1.686	0.074
Soft drink frequency					
Seldom^a^	25.88	209 (42.05)			
Sometimes	40.47	329 (42.34)	1.012	0.806-1.271	0.919
Frequently	33.65	289 (44.74)	1.116	0.881-1.413	0.364
Milk, tea, coffee with sugar					
Seldom^a^	29.89	233 (40.59)			
Sometimes	31.30	261 (44.85)	1.190	0.942-1.503	0.144
Frequently	39.79	333 (43.59)	1.131	0.908-1.408	0.273

^a^Reference category; ^b^binary logistic regression.

### Cumulative Multivariate Logistic Regression Results

Only statistically significant associations are presented. Residence (OR rural = 1.798, 95% CI: 1.485-2.177, p < 0.001), sex (OR female = 1.352, 95% CI: 1.121-1.631, p = 0.002), presence of calculus (OR yes = 1.279, 95% CI: 1.057-1.548), and snack consumption (OR frequently = 1.418, 95% CI: 1.064-1.890, p = 0.017) were risk factors, while paternal educational level (OR > 9 years = 0.755, 95% CI: 0.608-0.936, p = 0.011) was a protective factor (Table 7).

**Table 7:** Multivariate conditional Logistic regression analysis of factors related to permanent teeth caries among 12-year-old students in Guangdong Province

Variables	B	OR	95% CI	p-value
Residence (rural)	0.586	1.798	1.485-2.177	<0.001
Sex (female)	0.302	1.352	1.121-1.631	0.002
Paternal educational level (>9 years)	-0.281	0.755	0.608-0.936	0.011
Snack frequency (≥once a day)	0.350	1.418	1.064-1.890	0.017
Calculus (yes)	0.264	1.279	1.057-1.548	0.012

B = regression coefficient; OR = odds ratios; CI = confidence interval.

## Discussion

The decennial surveys collected data from examinations and questionnaires to assess multiple conditions associated with behaviours and attitudes. This study analysed systematic and large-scale data to assess the current oral health status among 12-year-olds in Guangdong according to the WHO criteria; the results provide high-quality data and have a high degree of lateral comparability to other regions. Moreover, we analysed various associated factors in the multivariate logistic regression analysis to identify possible risk factors for caries in permanent teeth and provided scientific evidence for the development of prevention strategies for policymakers and dental public health practitioners.

In this study, caries prevalence and DMFT (43.07%/ 1.06) were relatively high compared to the nationwide averages (38.5%/0.86).^33^ All of these values remain far from the oral health goal set by the Healthy China 2030 plan, which states that the prevalence of caries among 12-year-old children should be no more than 25%. The integration of Western culture has resulted in the adoption of Western diet patterns, and the wide availability of sugar due to rapid economic development and increased sugar production may have contributed to the high prevalence of caries in 12-year-olds in Guangdong in recent years; however, caries prevalence and DMFT were lower than those reported in other developing countries, including Quito, Ecuador (60.3%/1.61),^25^ and Brazil (56.0%/2.04).^12^ Over the past three decades, the average DMFT values in 12-year-old students in Guangdong were 0.99, 1.63, 0.54, and 1.06 in 1983, 1995, 2005, and 2015, respectively; these values were relatively low according to the WHO dental caries severity criteria.^33,36,37^ The sharp decline in caries between 1995 and 2005 may largely be due to changes in the diagnostic criteria and the fact that the CPI probe was used to reconfirm the presence of caries after a visual examination. Nevertheless, the prevalence of caries has decreased among children in developed countries, including England^27^ and the USA.^34^ Several factors, such as sampling methods, dietary habits, demographics, cultural differences, and oral health care systems, could be responsible for the different prevalence rates and severity of caries in different regions.

Regarding dental fluorosis, we identified a prevalence of 5.05% in Guangdong Province, which was lower than the national average prevalence rates in China (13.4%),^41^ Quito, Ecuador (63.7%),^25^ and the USA (65%).^28^ The CFI value (0.11) in this population indicates that there is currently no need for dental fluorosis intervention in Guangdong. The effective use of fluoride is one of the main factors responsible for the decline in the prevalence and severity of caries in most developed countries. No statistically significant associations between caries and fluorosis were observed in this study, although this topic needs further research.

In this study, we found that students in rural areas had higher caries prevalence rates than those in urban areas of Guangdong. This statistically significant difference can be explained by the inequality between urban and rural areas regarding economic status and medical resources. Although Guangdong is one of the most prosperous regions in China, medical resources are unevenly distributed and mostly concentrated in Guangzhou and Shenzhen. A similar residence-related difference in caries prevalence was found in the China national survey,^33^ in Korea,^21^ and in Poland,^14^ but a report from northwest Russia^16^ showed the opposite result. The differences between children from urban and rural areas could be explained by several factors. First, different dietary patterns and oral hygiene habits exist.^30^ Second, rural residents face obstacles in recognising the importance of oral health and obtaining timely treatment. These difficulties include a shortage of dental personnel, inadequate availability, a lack of accessibility to oral health services, and insufficient oral health knowledge and attitudes.^35^ Third, socioeconomic factors such as low household income and low parental education levels in rural areas might also account for these differences.^31^ In general, urban students have more opportunities to access oral health care and establish good oral hygiene than rural students.

Female students have a higher risk of caries than male students, which has been confirmed in this survey. This result has been well documented in epidemiological studies on caries in human populations. The sex difference in caries prevalence may be attributed to the earlier eruption of teeth in females, sex-based dietary preferences, and different saliva osmotic pressures. Another study involving clinical and experimental caries research confirmed the impact of hormonal fluctuations,^22,23^ which make the oral environment significantly more cariogenic in females during puberty, pregnancy, and menstruation than in males. The aetiology of caries is complex, and the quantity and quality of saliva constitute an important dimension in its occurrence and progression. Females showed a low salivary pH and similar flow rates compared to males in some studies,^13,22^ whereas other studies found the opposite.^11^ These findings revealed the complex and dynamic influences of physiological, behavioural, and environmental factors on sex differences in caries incidence.

Recent studies have shown that both paternal and maternal educational levels were inversely associated with caries in children.^8,20^ The results of this survey reported that almost half of the students’ fathers had a relatively low educational status (≤9 years); however, 55.8% of the students’ fathers had a university or higher level of education in a report involving a Saudi population.^1^ In addition, a high paternal educational level was a dominant protective factor. Interestingly, no statistically significant difference was found between the maternal educational levels in the multivariate logistic regression model. A strong correlation may exist between maternal and paternal education. Once other factors (diet, sex, etc) are considered, the mother’s influence might gradually disappear. The results may suggest that higher levels of education among fathers, but not necessarily mothers, reflect more household income. This may be explained by gender pay inequality in China.^32^ Moreover, highly educated parents are more willing than parents with a low educational level to spend time and money on developing good oral habits in children.^4^ Currently, most studies have focused on the association between caries and mothers’ educational backgrounds, with few studies discussing fathers’ attributes due to the assumption that mothers spend more time overseeing and have a greater influence on students’ behaviours than fathers. Little has been discussed about the impact of a father’s educational background on a student’s caries, which warrants future investigation.

The prevalence of calculus in this population (43.8%) was lower than that in Bulang, China (58%).^40^ Almost 50% of the students in this study reported brushing their teeth at least twice per day; however, over half of them were diagnosed with gingival bleeding. The results may be partly explained by the lack of oral education obtained by students and the lack of popularisation of proper brushing techniques. The presence of calculus was determined to be a risk factor for caries in 12-year-olds. Similar results were found in a study on caries risk prediction.^29^ In contrast, some researchers believe that calculus mineralisation potentially protects against caries.^9^ On the one hand, the presence of calculus indicates insufficient awareness of oral health in students and parents; on the other hand, calculus makes it difficult for students to maintain good oral hygiene, leading to long-term cariogenic substrates in the oral environment. These factors may increase the risk of caries. The relationship between the risk of caries in permanent teeth and the presence of calculus needs further research.

Caries and periodontal diseases are associated and share a common risk factor: the consumption of foods with added sugar.^6,24^ In our study, a high frequency of added sugar consumption (snacking) was a risk factor for caries among 12-year-old students in Guangdong Province. Numerous cross-sectional, epidemiological studies have documented a statistically significant correlation between caries and dietary sugar intake.^19,26^ The mean daily added sugar amount varies among children, teenagers, and adults. In the UK, the recommended sugar consumption for the total population is below 60 g/d. In Irish adults, the recommended added sugar intake frequency of four times per day corresponds to a mean added sugar intake of 9%, but for children and teenagers, this value exceeds the WHO recommendation.^19^ Previous studies have reported that the use of alternative sweeteners, clear and accurate labelling for added sugar-containing packaged foods and school-based methods for limiting the type of food offered by school cafeterias are worthwhile investments.^5,26,38^

The most obvious limitation of this study is its retrospective rather than prospective design, potentially introducing recall bias. An additional limitation is the lack of consideration of the water fluoride concentration in the study area, which may have a dominant impact on the prevalence in certain districts. Finally, the questionnaire administered in this study was self-reported, potentially resulting in several sources of partiality, especially participants’ memory bias.

## Conclusion

The prevalence of caries in permanent teeth was relatively high, and approximately half of the students had periodontal health problems in Guangdong Province. Rural residence, female sex, the presence of calculus, and frequent consumption of sugary snacks were the main risk factors for caries, while a high paternal education level was a protective factor. More preventive measures should be targeted especially at females, rural students, and those who had poor oral hygiene.
